# Distribution and dissemination of the Val1016Ile and Phe1534Cys *Kdr* mutations in *Aedes aegypti* Brazilian natural populations

**DOI:** 10.1186/1756-3305-7-25

**Published:** 2014-01-15

**Authors:** Jutta Gerlinde Birggitt Linss, Luiz Paulo Brito, Gabriela Azambuja Garcia, Alejandra Saori Araki, Rafaela Vieira Bruno, José Bento Pereira Lima, Denise Valle, Ademir Jesus Martins

**Affiliations:** 1 Laboratório de Fisiologia e Controle de Artrópodes Vetores, Instituto Oswaldo Cruz – FIOCRUZ, Rio de Janeiro, RJ, Brazil; 2 Laboratório de Entomologia, Instituto de Biologia do Exército, Rio de Janeiro, RJ, Brazil; 3 Laboratório de Biologia Molecular de Insetos, Instituto Oswaldo Cruz – FIOCRUZ, Rio de Janeiro, RJ, Brazil; 4 Instituto Nacional de Ciência e Tecnologia em Entomologia Molecular, Rio de Janeiro, RJ, Brazil; 5 Laboratório de Biologia Molecular de Flavivirus, Instituto Oswaldo Cruz - FIOCRUZ, Rio de Janeiro, RJ, Brazil

**Keywords:** *kdr* mutation, Pyrethroid resistance, Vector control, *Aedes aegypti*, Dengue in Brazil, Sodium channel

## Abstract

**Background:**

The chemical control of the mosquito *Aedes aegypti*, the major vector of dengue, is being seriously threatened due to the development of pyrethroid resistance. Substitutions in the 1016 and 1534 sites of the voltage gated sodium channel (AaNa_V_), commonly known as *kdr* mutations, confer the mosquito with knockdown resistance. Our aim was to evaluate the allelic composition of natural populations of Brazilian *Ae. aegypti* at both *kdr* sites.

**Methods:**

The AaNa_V_ IIIS6 region was cloned and sequenced from three Brazilian populations. Additionally, individual mosquitoes from 30 populations throughout the country were genotyped for 1016 and 1534 sites, based in allele-specific PCR. For individual genotypes both sites were considered as a single locus.

**Results:**

The 350 bp sequence spanning the IIIS6 region of the *AaNa*_*V*_ gene revealed the occurrence of the *kdr* mutation Phe1534Cys in Brazil. Concerning the individual genotyping, beyond the susceptible wild-type (Na_V_^S^), two *kdr* alleles were identified: substitutions restricted to the 1534 position (Na_V_^R1^) or simultaneous substitutions in both 1016 and 1534 sites (Na_V_^R2^). A clear regional distribution pattern of these alleles was observed. The Na_V_^R1^*kdr* allele occurred in all localities, while Na_V_^R2^ was more frequent in the Central and Southeastern localities. Locations that were sampled multiple times in the course of a decade revealed an increase in frequency of the *kdr* mutations, mainly the double mutant allele Na_V_^R2^. Recent samples also indicate that Na_V_^R2^ is spreading towards the Northern region.

**Conclusions:**

We have found that in addition to the previously reported Val1016Ile *kdr* mutation, the Phe1534Cys mutation also occurs in Brazil. Allelic composition at both sites was important to elucidate the actual distribution of *kdr* mutations throughout the country. Studies to determine gene flow and the fitness costs of these *kdr* alleles are underway and will be important to better understand the dynamics of *Ae. aegypti* pyrethroid resistance.

## Background

Dengue is currently the most important arbovirus in the world. Dengue has spread widely in urban areas of tropical and subtropical regions during the last decades, including countries of Southeast Asia, Pacific and Latin America [1]. Between 2001–2011, almost 10 million dengue cases were reported in Latin America, almost 60% of these were registered in Brazil [2]. Dengue mortality can reach up to 5% of the confirmed infection cases. In addition, in tropical dengue endemic countries a loss of 1,300 disability-adjust life years (DALYs) per million people is estimated [1]. *Aedes aegypti* is the main dengue vector throughout the world. Control of this mosquito consists primarily of the elimination of artificial and disposable water flooded larvae breeding sites and application of insecticides. The WHO Pesticide Evaluate Scheme (WHOPES) recommends ten different compounds to eliminate larvae, including neurotoxicants (organophosphates, pyrethroids and neocotinoids), Insect Growth Regulators (chitin synthesis inhibitors and juvenile hormone analogs), and *Bacillus* (like *B. thuringiensis var israelensis*) as larvicides. However, fewer formulations are recommended for the control of adult mosquitoes, mostly five pyrethroids and one organophosphate [3].

Given their rapid mode of action and low hazardous effect to the environment, compared to organophosphate insecticides, the use of pyrethroids has increased significantly in the last two decades. Nowadays, pyrethroids are widely employed in and around households, even for pet protection and mosquito control [4]. Since *Ae. aegypti* is essentially an urban mosquito, it is constantly exposed to strong pyrethroid selection. As a consequence, many *Ae. aegypti* populations worldwide are becoming resistant to this class of insecticides [5].

Pyrethroids target the transmembrane voltage gated sodium channel (Na_V_) from the insect nervous system, triggering rapid convulsions followed by death, a phenomenon known as *knockdown* effect [6]. The Na_V_ is composed of four homologous domains (I-IV), each with six hydrophobic segments (S1-S6) [7]. Because the Na_V_ is a very conserved protein among invertebrates, small changes are permissive without impairing its physiological role [8]. A series of mutations have been identified in different orders of insects and acarids that affect pyrethroid susceptibility, thus being referred to as ‘*k*nock*d*own *r*esistance’ or *kdr* mutations [9]. These *kdr* mutations may lead to conformational changes in the whole channel that maintain its physiological role but avoid insecticide action [10].

In insects, the most common *kdr* mutation is the substitution Leu/Phe in the 1014 site (numbered according to the *Musca domestica* Na_V_ primary sequence), followed by the Leu/Ser substitution in the same position, in *Anopheles* and *Culex* mosquitoes [11]. In the *Ae. aegypti* Na_V_ (*AaNa*_*V*_), the 1014 Leu codon is encoded by CTA, rather than TTA as in *Anopheles* and *Culex* mosquitoes. This means that two substitutions would have to be simultaneously selected in the same codon in order to change Leu to Phe (TTT) or Ser (TCA) [12]. Although several mutations have been identified in natural populations at *AaNa*_*V*_[13], only the Val1016Ile and Phe1534Cys substitutions were clearly related to the loss of pyrethroid susceptibility [12,14]. These sites are placed respectively in the IIS6 and IIIS6 regions of the channels that are known to be involved in the interaction with pyrethroids [10]. It has been previously observed that in Latin America the 1016 Ile *kdr* is highly disseminated [12,15,16] and its frequency is rapidly increasing in localities with intense pyrethroid use, such as Brazil and Mexico [15,16]. High frequencies of 1534 Cys *kdr* were also observed in Grand Cayman and Martinique [14,17].

In the current study, we demonstrate that the1534 Cys *kdr* mutation is present in Brazil together with the 1016 Ile allele previously found. The simultaneous occurrence of both *kdr* mutations at the 1016 and 1534 was found in several localities. Spatial and temporal analysis of these alleles point to a significant role of the *kdr* mutations in pyrethroid resistance in Brazil.

## Methods

### Mosquito samples

*Ae. aegypti* used for *kdr* genotyping originated from the same samples evaluated by the Brazilian *Aedes agypti* Insecticide Resistance Monitoring Network, collected with ovitraps according to recommendations of the Brazilian Dengue Control Program [18]. Adult mosquitoes resulting from the eggs collected in the field (F0 generation) were preferentially used. However, in some cases only the following generations reared in the laboratory were available. Details regarding sampling as well as individual data from mosquitoes used for *kdr* genotyping are found in Table 1. A total of 30 localities were analyzed at least once, with AJU, SGO, MSR and VIT analyzed for two-four time-points.

**Table 1:** ***Aedes aegypti *** **populations used in this study**

**Code**	**Municipality**	**Locality state**	**Coordinates**	**Brazilian macroregion**	**Year of sampling**	**Generation used in the assays**	**Gender**
AJU	Aracajú	Sergipe	10°54*'*AJU S, 37°04*'*O	Northeast	2002	F1	Males
					2006	F1	Females
					2010	F1	Females
					2012	F0	Males
APG	Aparecida de Goiânia	Goiás	16°48*'*S, 49°14*'*O	Central-west	2012	F0	Males
BEL	Belém	Pará	1°27*'*S, 48°30*'*O	North	2010	F1	Males
BVT	Boa Vista	Roraima	2°49*'*N, 60°40*'*O	North	2011	F1	Males
CAC	Caicó	Rio Grande do Norte	6°27*'*S, 37°05*'*O	Northeast	2010	F1	Females
CAS	Castanhal	Pará	1°17*'*S, 47°55*'*O	North	2011	F0	Males
CBL	Campos Belos	Goiás	13°02*'*S, 46°45*'*O	Central-west	2011	F0	Males
CGR	Campo Grande	Mato Grosso do Sul	20°26*'*S, 54°38*'*O	Central-west	2010	F0	Males
CIT	Cachoeiro do Itapemirim	Espírito Santo	20°51*'*S, 41°06*'*O	Southeast	2012	F0	Males
CLT	Colatina	Espírito Santo	19°32*'*S, 40°37*'*O	Southeast	2011	F0	Males
DQC	Duque de Caxias	Rio de Janeiro	22°47*'*S, 43°18*'*O	Southeast	2001	F3	Females
					2010	F1	Males
					2012	F0	Males
FOZ	Foz do Iguaçú	Paraná	25°32*'*S, 54°35*'*O	South	2009	F2	Females
GVD	Governador Valadares	Minas Gerais	18°50*'*S, 41°56*'*O	Southeast	2011	F1	Males
ITP	Itaperuna	Rio de Janeiro	21°12*'*S, 41°53*'*O	Southeast	2011	F2	Males
LZN	Luziânia	Goiás	16°15*'*S, 47°55*'*O	Central-west	2011	F2	Females
MRB	Marabá	Pará	5°22*'*S, 49°07*'*O	North	2011	F0	Males
MSR	Mossoró	Rio Grande do Norte	5°11*'*S, 37°20*'*O	Northeast	2009	F0	Males
					2011	F0	Males
PCR	Pacaraima	Roraima	4°25*'*N, 61°08*'*O	North	2011	F0	Males
PGT	Porangatu	Goiás	13°25*'*S, 49°08*'*O	Central-west	2012	F0	Males
PNM	Parnamirim	Rio Grande do Norte	5°54*'*S, 35°15*'*O	Northeast	2010	F0	Males
RVD	Rio Verde	Goiás	17°47*'*S, 50°55*'*O	Central-west	2011	F0	Males
SGO	São Gonçalo	Rio de Janeiro	22°49*'*S, 43°03*'*O	Southeast	2002	F2	Males
					2008	F2	Males
SIP	Santana do Ipanema	Alagoas	9°21*'*S, 37°14*'*O	Northeast	2010	F2	Maless
SMA	São Miguel do Araguaia	Goiás	13°15*'*S, 50°09*'*O	Central-west	2012	F0	Males
SRO	Santa Rosa	Rio Grande do Sul	27°52*'*S, 54°28*'*O	South	2011	F1	Males
SSO	São Simão	Goiás	18°59*'*S, 50°32*'*O	Central-west	2011	?	Males
STR	Santarém	Pará	2°26*'*S, 54°41*'*O	North	2010	F0	Males
TCR	Tucuruí	Pará	3°46*'*S, 49°40*'*O	North	2010	F0	Males
URU	Uruaçu	Goiás	14°31*'*S, 49°09*'*O	Central-west	2011	F0	Males
VIT	Vitória	Espírito Santo	20°18*'*S, 40°18*'*O	Southeast	2006	F1	Males
					2010	F0	Males

### Genotyping assays

Thirty individual mosquitoes from each locality were genotyped at both 1016 and 1534 positions from genomic DNA by allele-specific PCR (AS-PCR) which contains a common primer and two specific primers targeting each polymorphic site. The specificity is attained in the 3′-end, strengthened by a transition three nucleotides before [19]. Additionally, a GC-tail of different sizes was added at the 5′-end of these primers so products can be distinguished by their melting temperature (Tm) in a melting curve analysis or by electrophoresis [12,20,21]. Primer sequences are shown in Table 2. DNA extraction and amplification of the 1016 (Val/Ile) site were conducted as previously described [15]. The reaction for the 1534 (Phe/Cys) site was optimized from previous work [16,22]. In both cases, PCR was carried out with the GoTaq Green Master Mix kit (Promega), 0.5 μL of genomic DNA, 0.24 μM of the common primer, 0.12 and 0.24 μM of the specific primers (1534 Cys^*kdr*^ and 1534 Phe), in a total volume of 12.5 μL. Denaturing, annealing and extension conditions were, respectively, 95°C ⁄ 30″, 54°C ⁄ 40″ and 72°C ⁄ 45″, in 32 cycles. Alternatively, real-time PCR was conducted with the SYBR Green PCR Master Mix kit (LifeTechnologies/Applied Biosystems), 1 μL genomic DNA and 0.24 μM of each primer, in a total volume of 10 μL. The best conditions for denaturing, annealing and extension were respectively 95°C ⁄ 15″, 54°C ⁄15″ and 60°C ⁄ 30″, in 33 cycles, followed by a standard melting curve stage. The amplification reaction and melting curve analyses were performed in a StepOne Plus or in a 7500 Real-time PCR system (LifeTechnologies/Applied Biosystems). DNA pools of individuals from CGR, STR and PNM were used to amplify the region spanning the Na_V_ IIIS6 segment with the primers AaEx31P and AaEx31Q (Table 2), as specified elsewhere [14]. The PCR products were purified in S-400 microcolumns (GE Healthcare) according to manufacturer instructions and cloned with CloneJet PCR Cloning Kit (Thermo Scientific). The DNA sequencing was carried out in an ABI377 Sequencer with the Big Dye 3.1 Kit (LifeTechnologies/Applied Biosystems). Sequence analysis was performed using the BioEdit software version 7.2.

**Table 2:** Primer sequences

**Primer name**	**Sequence (5′ - 3′)**	**References**
1016 Val^+^ (for)	^##^ACAAATTGTTTCCCACCCGCAC*C*GG	[12,15]
1016 Ile^*kdr*^ (for)	^#^ACAAATTGTTTCCCACCCGCAC*T*GA	
1016 comom (rev)	GGATGAACCGAAATTGGACAAAAGC	
1534 Phe^+^ (for)	^#^TCTACTTTGTGTTCTTCATCAT*A*TT	[22]
1534 Cys^*kdr*^ (for)	^##^TCTACTTTGTGTTCTTCATCAT*G*TG	
1534 comom (rev)	TCTGCTCGTTGAAGTTGTCGAT	
AaEx31P (for)	TCGCGGGAGGTAAGTTATTG	[14]
AaEx31Q (rev)	GTTGATGTGCGATGGAAATG	
long 5*'*-tail	GCGGGCAGGGCGGCGGGGGCGGGGCC	
short 5*'*-tail	GCGGGC	

^+^wild-type specific primer, ^*kdr*^*kdr* specific primer, ^#^short 5′tail attached, ^##^long 5′tail attached.

All individuals were genotyped for both 1016 and 1534 sites. Linkage disequilibrium was tested by the online Genepop version 4.2 [23], and since the 1016 and 1534 sites are linked (see Results section), genotypic and allelic frequencies were taken as a single locus. Hardy-Weinberg equilibrium was evaluated by the classical equation [24], being the null hypothesis of equilibrium checked by a chi-square test with three or one degrees of freedom, respectively, when six or three genotypes were evidenced.

## Results

### Allele-specific discrimination

**Figure 1:**
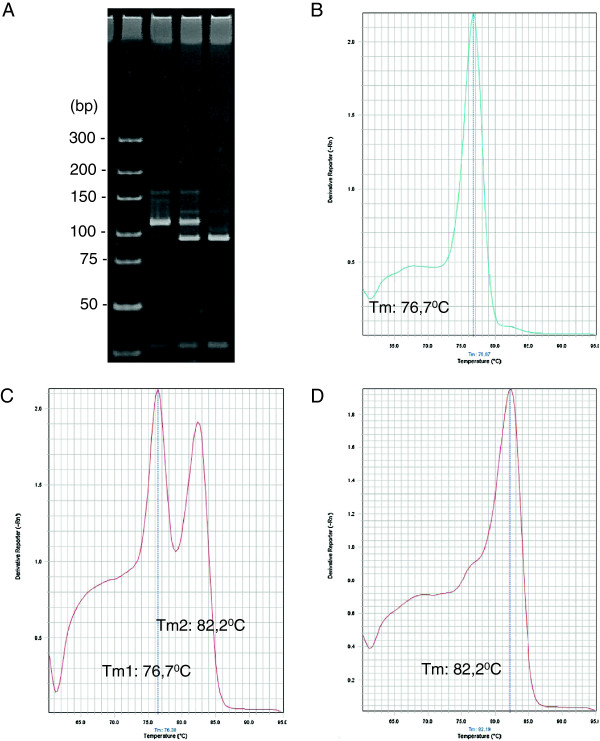
**Allele specific PCR (AS-PCR) for genotyping *kdr *mutations in the *Aedes aegypti *voltage gated sodium channel.** All panels represent reactions for the 1534 site. **(A)** Visualization of the amplicons in a 10% polyacrylamide gel electrophoresis, run under 170 V/45*'* and stained with ethidium bromide (1 μg/mL). Amplicons of approximately 90 and 110 bp correspond to alleles 1534 Phe^+^ and 1534 Cys^*kdr*^, respectively. DNA ladder was used as size marker (O’GeneRuler DNA Ladder, Ultra Low Range/Fermentas, 150 ng). Dissociation curve analysis in real time PCR differentiating the Phe/Phe **(B)**, Phe/Cys **(C)**, and Cys/Cys **(D)** genotypes. The Tm for the respective alleles are indicated.

A 20 bp size difference, due to the 5′-GC tail of allele specific primers, enabled the easy discrimination of homozygous and heterozygous genotypes in either a polyacrylamide gel electrophoresis or in dissociation curves through real-time PCR (Figure 1). Electrophoresis revealed products of around 80 and 100 bp, respectively for Ile^*kdr*^ and Val^+^ (1016 reaction), and 90 and 110 bp, respectively for Phe^+^ and Cys^*kdr*^ (1534 reaction). The dissociation curve exhibited Tm of around 76 and 84°C, respectively for Ile^*kdr*^ and Val (1016 reaction), and 77 and 82°C, respectively for Phe and Cys^*kdr*^ (1534 reaction). The PCR conditions of annealing temperature, number of cycles and concentration of each primer were crucial to avoid unspecific amplification. All reactions were accompanied by positive controls, each one consisting of the three potential genotypes at the 1016 and 1534 positions, which were obtained by previously genotyped individuals: homozygous wild type, heterozygous, and homozygous *kdr*. As the Phe1534Cys mutation was detected for first time in Brazilian samples, we cloned and sequenced the IIIS6 region (exon 31) of the *AaNa*_*V*_ gene of three genotyped populations (CGR, STR and PNM), confirming the primers’ specificity. The 350 bp fragments were submitted to GenBank (accession numbers: KF527414 and KF527415, for 1534 Cys^*kdr*^ and 1534 Phe^+^, respectively). Excluding the site of the 1534 *kdr* mutation (TTC/TGC), no other polymorphic site was detected relative to the sequence deposited in VectorBase (Liverpool strain).

### Genotyping 1016 and 1534 *Aa*Na_V_ sites in natural populations

**Figure 2:**
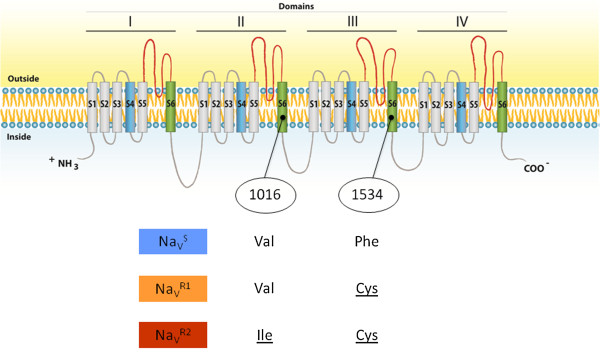
**Voltage gated sodium channel and the 1016 and 1534 alleles found in Brazilian *Aedes aegypti *populations.** The Na_V_ is represented with its four domains (I-IV), each with the six transmembrane segments (S1-S6). The voltage sensitive S4 and the pore forming S6 segments are colored in blue and green, respectively (scheme adapted from [9]). The 1016 and 1534 *kdr* sites in *Aedes aegypti* are indicated. Mutant amino acids are underlined.

**Figure 3:**
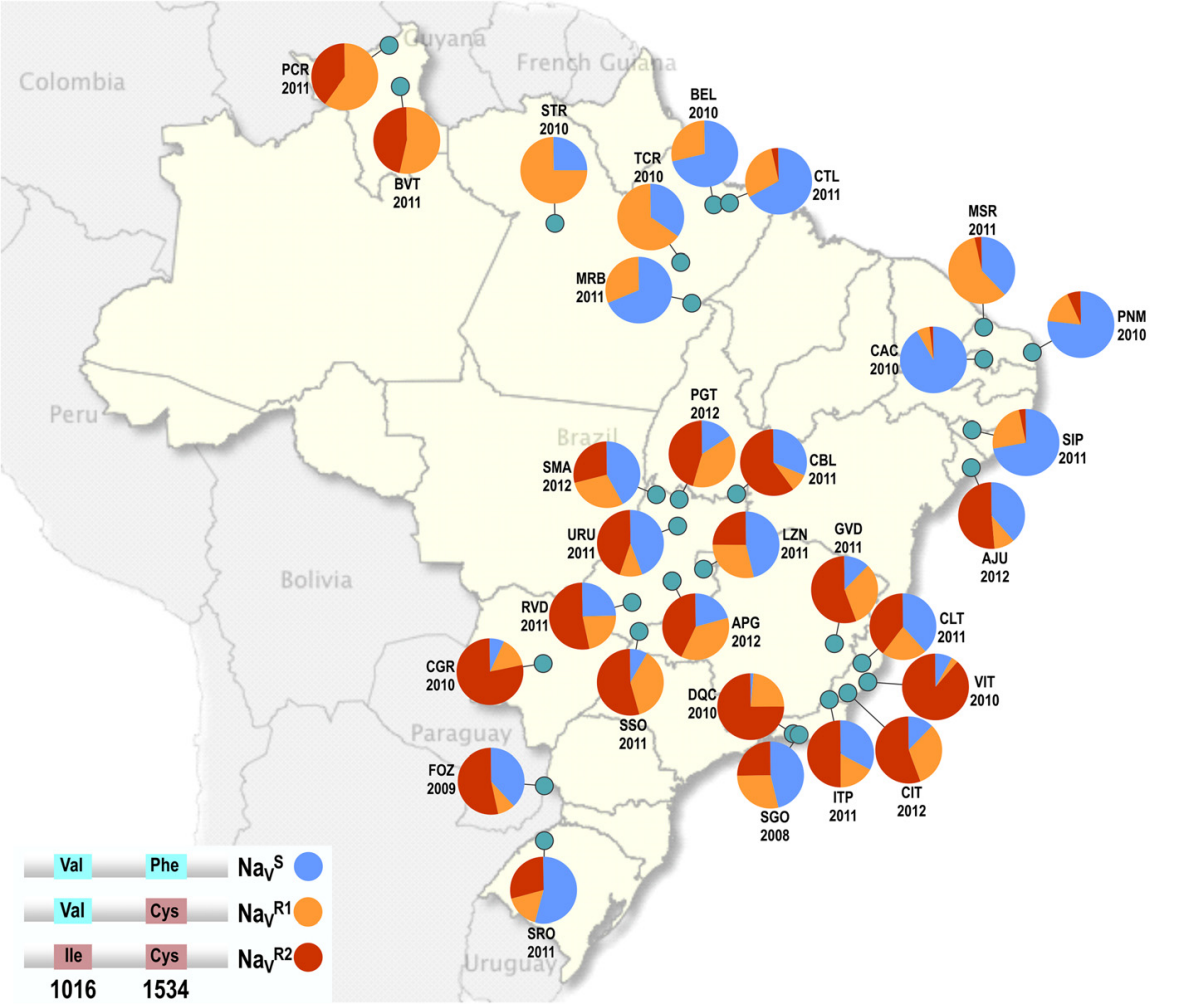
**Distribution of the *kdr *alleles in Brazilian *Aedes aegypti *populations.** For each locality, only the most recent samples evaluated are shown. Details of the localities are shown in Table 1. Alleles are represented according to the colors used in Figure 2.

Around 30 *Ae. aegypti* individuals from each one of 30 distinct Brazilian localities were genotyped for both 1016 and 1534 Na_V_ sites, totalling 1,112 analyzed mosquitoes. Some localities were sampled two to four times within a ten-year interval. The genotypes of individual mosquitoes for both sites were first calculated independently: 1016 Val^+^/Val^+^, Val^+^/Ile^*kdr*^ and Ile^*kdr*^/Ile^*kdr*^, and 1534 Phe^+^/Phe^+^, Phe^+^/Cys^*kdr*^ and Cys^*kdr*^/Cys^*kdr*^. These data were used to perform a genotypic linkage disequilibrium analysis and total linkage between them was demonstrated (Fisher’s method, p < 0.001), as expected from two sites placed in the same gene. In this sense both sites were considered as constituents of a single locus, thus evidencing the occurrence of six genotypes in individual mosquitoes (Table 3). Based on the composition of these genotypes, we concluded that three alleles were present in the evaluated samples: ‘1016 Val^+^ + 1534 Phe^+^’ (wild-type), ‘1016 Val^+^ + 1534 Cys^*kdr*^’ (1534 *kdr*) and ‘1016 Ile^*kdr*^ + 1534 Cys^*kdr*^’ (1016 *kdr* + 1534 *kdr*). Hereafter these alleles will be simply referred to as ‘Na_V_^S^’, ‘Na_V_^R1^’ and ‘Na_V_^R2^’, respectively (Figure 2). Double mutants and individuals with mutation only in the 1534 position were found (respectively, Na_V_^R2^ and ‘Na_V_^R1^); however, in no case was the 1016 *kdr* mutation observed alone, precluding the existence of a 1016 Ile^*kdr*^ + 1534 Phe^+^ allele in the evaluated populations. Figure 3 shows the frequencies for Na_V_^S^, Na_V_^R1^ and Na_V_^R2^ alleles in the most recent samples obtained from each locality. The 95% CI of the allele frequencies is shown in the Additional file 1: Table S1. According to the alleles, the genotypes were named SS, SR1, SR2, R1R1, R1R2 and R2R2. Their frequencies and the Hardy-Weinberg Equilibrium deviation test are presented in Table 3. In only seven out of 38 samplings the Hardy-Weinberg Equilibrium assumption was rejected (p < 0.05). No specific genotype contributed to the deviation in these seven localities.

**Table 3:** **Genotype frequencies of Brazilian *Aedes aegypti *** **populations at the 1016 and 1534 sites of the Na**_**V **_**locus**

**Macro-region**	**Population**	**Genpotype frequencies**						**Total (n)**	**HWE test**	
		**SS**	**SR1**	**SR2**	**R1R1**	**R1R2**	**R2R2**		**χ**^**2**^	**p**
North	PCR11	0.000	0.000	0.000	0.367	0.467	0.167	30	0.0	0.879
	BVT11	0.000	0.000	0.000	0.536	0.393	0.071	28	0.0	0.993
	CAS11	0.400	0.500	0.033	0.033	0.033	0.000	30	2.3	0.512
	BEL10	0.536	0.357	0.000	0.107	0.000	0.000	28	0.4	0.932
	STR10	0.200	0.100	0.000	0.700	0.000	0.000	30	16.1	0.000
	TCR10	0.200	0.300	0.000	0.500	0.000	0.000	30	3.5	0.062
	MRB11	0.621	0.138	0.000	0.241	0.000	0.000	29	13.3	0.000
Northeast	MSR09	0.600	0.367	0.000	0.033	0.000	0.000	30	0.2	0.660
	MSR11	0.000	0.767	0.000	0.200	0.000	0.033	30	14.9	0.002
	PNM10	0.704	0.111	0.037	0.111	0.037	0.000	27	9.3	0.025
	CAC10	0.833	0.133	0.033	0.000	0.000	0.000	30	0.0	0.998
	SIP10	0.433	0.500	0.067	0.000	0.000	0.000	30	4.7	0.199
	AJU02	1.000	0.000	0.000	0.000	0.000	0.000	30	0.0	1.000
	AJU06	0.767	0.033	0.167	0.000	0.033	0.000	30	0.3	0.955
	AJU10	0.269	0.038	0.308	0.000	0.000	0.385	26	3.6	0.306
	AJU12	0.200	0.033	0.333	0.033	0.100	0.300	30	3.4	0.338
Central-west	CBL11	0.069	0.069	0.414	0.000	0.103	0.345	29	0.5	0.918
	SMA12	0.207	0.172	0.241	0.103	0.207	0.069	29	1.2	0.750
	PGT12	0.000	0.069	0.241	0.241	0.241	0.207	29	5.9	0.115
	URU11	0.233	0.133	0.300	0.000	0.100	0.233	30	1.3	0.723
	LZN11	0.200	0.333	0.200	0.033	0.167	0.067	30	1.7	0.639
	APG12	0.000	0.207	0.207	0.138	0.241	0.207	29	2.5	0.466
	RVD11	0.103	0.034	0.241	0.069	0.241	0.310	29	2.8	0.421
	SSO11	0.000	0.133	0.033	0.200	0.233	0.400	30	7.6	0.056
	CGR10	0.000	0.033	0.100	0.000	0.267	0.600	30	1.2	0.749
Southeast	GVD11	0.000	0.033	0.200	0.267	0.067	0.433	30	18.3	0.000
	CLT11	0.067	0.333	0.300	0.000	0.100	0.200	30	9.0	0.029
	VIT06	0.267	0.100	0.333	0.000	0.033	0.267	30	2.4	0.492
	VIT10	0.000	0.067	0.100	0.000	0.000	0.833	30	2.3	0.507
	CIT12	0.000	0.069	0.138	0.103	0.172	0.517	29	3.8	0.281
	ITP11	0.148	0.111	0.259	0.074	0.074	0.333	27	5.0	0.172
	SGO02	1.000	0.000	0.000	0.000	0.000	0.000	30	0.0	1.000
	SGO08	0.192	0.231	0.308	0.115	0.115	0.038	26	1.6	0.669
	DQC01	1.000	0.000	0.000	0.000	0.000	0.000	30	0.0	1.000
	DQC10	0.000	0.033	0.067	0.100	0.067	0.733	30	13.0	0.005
	DQC12	0.000	0.033	0.000	0.000	0.433	0.533	30	5.6	0.136
South	FOZ09	0.133	0.100	0.400	0.033	0.000	0.333	30	3.6	0.311
	SRO11	0.296	0.259	0.222	0.037	0.000	0.185	27	7.4	0.059

Overall, the distribution of the three alleles differed according to the geographical region (Figure 3). In the North and Northeast Regions, the Na_V_^R1^ allele, mutant only at position 1534, was found in all localities, nevertheless the Na_V_^S^ wild-type allele was the most representative in six of the localities (BEL, CTL, MRB, CAC, SIP and PNM). The highest frequency of Na_V_^R1^, was found in the North: 0.750 (STR), among all populations analyzed. On the other hand, with exception of the most recent AJU (AJU2012), the Na_V_^R2^ double mutant allele was either absent or < 5% in the North and Northeast of Brazil. In contrast, the wild-type allele, Na_V_^S^, was absent from the two northernmost localities evaluated (PCR and BVT, both in the State of Roraima), where both mutant alleles were at high frequencies. In all localities from Central-West, Southeast and South regions, all three alleles were present. The most frequent allele was the Na_V_^R2^ double mutant. Exceptions were LZN, SMA, URU, SGO and SRO, where the Na_V_^S^ wild-type allele was the most representative (Figure 3).

**Figure 4:**
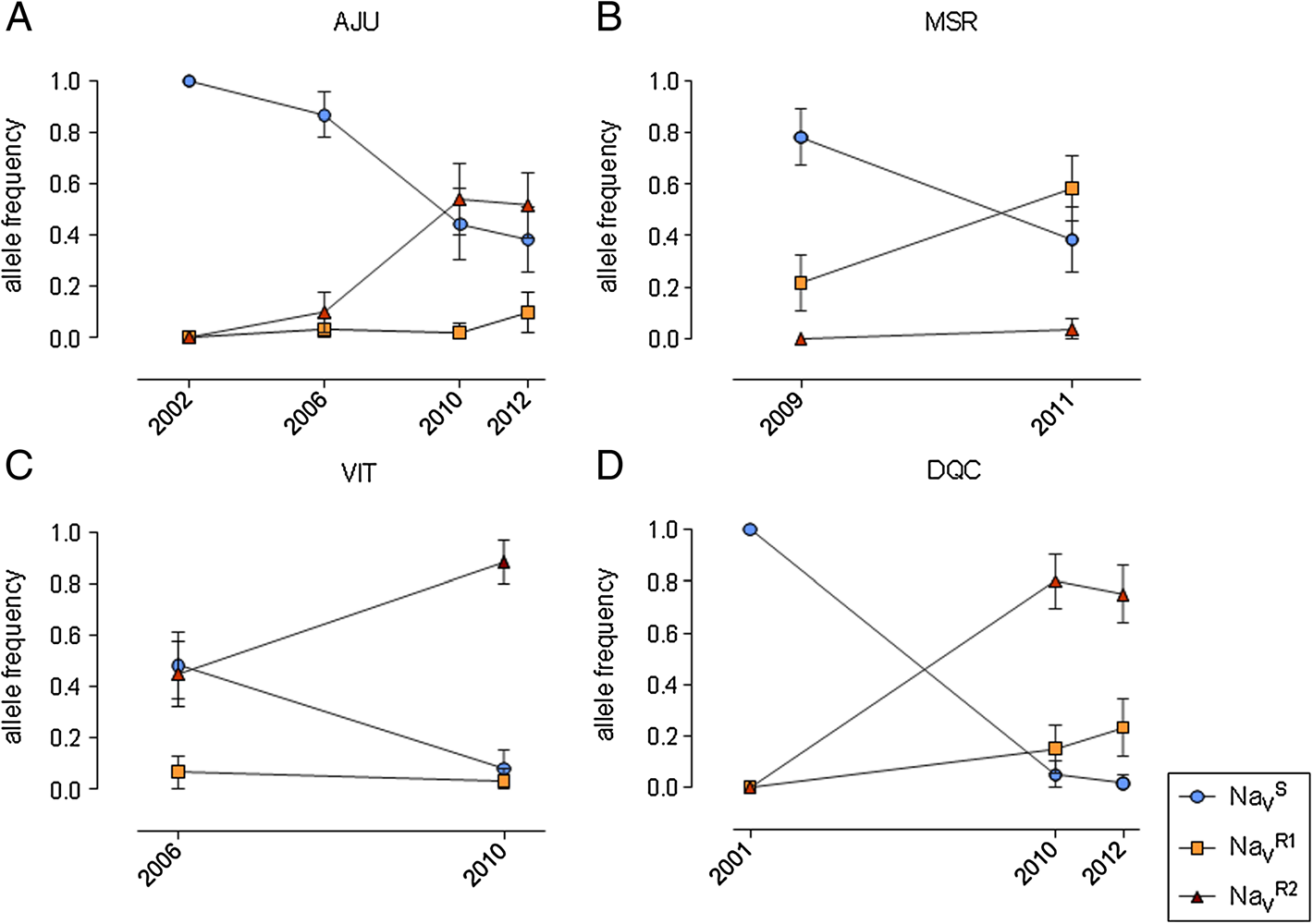
**Time-course of *kdr *alleles frequencies in four Brazilian *Aedes aegypti *populations.** Localities: **A** - Aracaju (AJU), **B** - Mossoró (MSR), **C** - Vitória (VIT) and **D** - Duque de Caxias (DCQ). Bars indicate the 95% CI of allele frequencies.

The dynamics of the genotype frequencies was analyzed in AJU, MSR, VIT and DQC. Samples from AJU were collected four times in the course of a decade, between 2002 and 2012. In 2002, only the Na_V_^S^ wild-type allele was detected. The *kdr* alleles appeared first in 2006 and the double mutant Na_V_^R2^ was the most frequent allele by 2012 (Figure 4). Accordingly, the ‘SS’ wild-genotype progressively decayed from 100% in 2002 to 20% in 2012, when the double mutant ‘R2R2’ represented 30% of the individuals, and was the most frequent genotype (AJU2012, Table 3). The frequency of the Na_V_^S^ wild-type allele also decreased in all other localities evaluated where the *kdr* alleles increased in frequency (Figure 4). Except for MSR, the Na_V_^R2^ double mutant is likely to be the most favorably selected allele. It is noteworthy that in AJU, the Na_V_^R1^ allele showed the larger frequency increase, probably because Na_V_^R2^ must have arrived to the Northeast more recently.

## Discussion

The genotyping of mutations directly related to insecticide resistance is an important surveillance tool for agricultural and sanitary purposes. Among selected mechanisms of pyrethroid resistance, *kdr* mutations in the voltage gated sodium channel (Na_V_) are those that better correlate particular genotypes with insecticide resistance [25]. The increased efficiency of insecticide detoxification, known as metabolic resistance – involving super families of enzymes such as GST, esterases and especially the multi function oxidases P450 – may also confer resistance to pyrethroids. However, identification of these mechanisms is mainly based on enzymatic assays of low specificity [26] or on bioassays with synergist compounds [27], and are not clearly linked to particular genes. More recently, many successful transcriptome tools for metabolic resistance genes have emerged, pointing to a very complex and diverse scenario regarding insecticide selected genes and their pattern of expression among insect populations [28,29]. Because the metabolic resistance based selection seems to have a high fitness cost, due to reallocation of energetic resources, this mechanism is expected to induce lower resistance levels, if compared to mutations in the target site molecules [30]. This was corroborated by laboratory selection with pyrethroids in an *Ae. aegypti* lineage: increase of the 1016 Ile^*kdr*^ frequency was inversely proportional to the number of ‘metabolic’ genes differentially transcribed [29]. It was hypothesized that, in the presence of pyrethroid, *kdr* mutations are preferentially selected among other mechanisms, contributing to higher resistance levels and/or resulting in less deleterious effects.

In addition to the classical Leu1014Phe *kdr* mutation, several others have been associated with pyrethroid resistance [6]. Interactions of multiple Na_V_ mutations may modulate pyrethroid resistance levels. For instance, certain Na_V_ haplotypes, including synonymous substitutions, were found in two distinct field populations of *Culex quinquefasciatus* selected for pyrethroid resistance during 6–8 generations in the laboratory. It was suggested that some of these haplotypes were selected at an early stage of permethrin resistance and later evolved to other mutation combinations in the course of selection pressure [31]. In *Ae. aegypti*, a synonymous substitution at exon 20, together with an extensive polymorphism in the following intron, were linked to both Ile1011Met and Val1016Ile mutations [15,32]. Additionally, a gene duplication event was recently described in the *AaNa*_*V*_ of natural populations and in a laboratory strain selected for pyrethroid resistance [33]. Although there are at least seven different mutations described in the *AaNa*_*V*_, only those corresponding to the 1016 and 1534 positions are clearly related to resistance; both are placed in a domain of the sodium channel that interacts directly with the pyrethroid molecule [34].

There are two mutations described in the *Aa*Na_V_ 1016 site, Val to Ile or Gly, respectively in Latin America [12,14,15] and in Southeast Asia [35]. In Brazil, we found no evidence of a haplotype that contains exclusively the 1016 Ile^*kdr*^ mutation, since it was always found together with 1534 Cys^*kdr*^ (Na_V_^R2^ allele, herein). Nevertheless, we are aware that it is possible for a haplotype carrying the 1016 *kdr* mutation to occur in the populations examined, however, it would be present at very low frequencies. Actually, this putative allele must have occurred in two out of three *Ae. aegypti* populations from Grand Cayman, given that the 1016 Ile^*kdr*^ presented a higher frequency than the 1534 Cys^*kdr*^ substitution [14].

Differently from the 1016 position, only one substitution, Phe/Cys, was found in the 1534 site by far [14,36]. This 1534 substitution can be linked with another one. In Thailand, the 1534 Cys^*kdr*^ co-occurred with 1016 Gly^*kdr*^ and 989 Pro^*kdr*^ in the same molecule [22]. In that region an allele 1534 Cys^*kdr*^ without mutation in 1016 site (Na_V_^R1^ allele, herein) seemed to be very common, since its frequency was higher than the 1016 Gly^*kdr*^[35].

Here we presented the distribution of the *kdr* variants for the AaNa_V_, considering both 1016 and 1534 sites screen from several natural Brazilian populations. We considered that once these sites are very close in the genome, reporting the allele/genotypic frequencies of each site separately would not be fully informative. However, because there are still some gaps concerning the actual role of these mutations in pyrethroid resistance, regarding whether they are acting alone or synergistically, and present in *cis* or trans mutations, we are reporting the allele frequencies of each site rather than as an haplotype. The implication of the 1016 Ile^*kdr*^ allele in resistance to pyrethroids was corroborated by laboratory selection, which highly increased the allelic frequency up to fixation in only five generations [29]. Accordingly, in the last decade this mutation has been rapidly spreading in natural populations from Brazil and Mexico, concomitantly with the intensification of pyrethroid usage due to the emergence of severe dengue outbreaks [15,16]. In these cases however, the co-occurrence of the 1534 Cys^*kdr*^ mutation has been overlooked. A recent study reported high frequencies of 1534 Cys^*kdr*^ in Grand Cayman [14], suggesting it is not a novel mutation in Latin America. In a recent report, nine single and two double *AaNa*_*V*_ mutants were constructed and inserted in a *Xenopus* oocyte system in order to perform functional evaluations of these substitutions in the presence of type I or II pyrethroids [34]. The 1016 Ile^*kdr*^ construct did not result in sensitivity reduction, to either pyrethroid types. On the other hand, the 1534 Cys^*kdr*^ significantly diminished the AaNa_V_ sensibility to type I but not to type II pyrethroids. This same substitution in the homologous *kdr* site of the cockroach Na_V_ exhibited similar results [37].

An *Ae. aegypti* lineage, selected for permethrin resistance in the laboratory, exhibited high frequencies of 1016 Gly^*kdr*^ + 1794 Tyr^kdr^ substitutions in the same molecule, which suggested a synergistic effect towards pyrethroid resistance [38]. We hypothesize that mutation in the 1016 site should be important when in synergism with other specific mutations. In Brazil, the 1534 Cys^*kdr*^ mutation is widespread throughout the territory. The Na_V_^R1^ allele is more frequent in North/Northeast regions whereas Na_V_^R2^ is more commonly present in Central/Southeast regions, generally where the highest resistance levels to pyrethroids are observed [18]. Both mutant haplotypes appear to be rapid and favorably selected in all evaluated populations. However, in the most recent samplings the Na_V_^R2^ double mutant was the more frequent *kdr* allele. The exception was MSR, in the Northeast Region, where Na_V_^R2^ was only recently introduced. Together these data suggest that Na_V_^R2^ allele would be more advantageous for pyrethroid resistance, or impose a lower fitness cost when compared to Na_V_^R1^. We recently demonstrated that an Na_V_^R2^ homozygous *Ae. aegypti* lineage, highly resistant to pyrethroids, exhibited a fitness cost in a series of life-trait parameters [39]. Further comparisons between Na_V_^R1^ and Na_V_^R2^ lineages will be of importance to better clarify those assumptions.

It is of note that since 2001 and up to 2009 the Brazilian Dengue Control Program employed pyrethroids in ultralow volume applications in several municipalities as part of the effort to control the dengue vector [18]. With very few exceptions, the basis for pyrethroid selection pressure derived from national campaigns is essentially the same in the whole country. Therefore, differential selection pressures would not explain the aforementioned regionalization of the *kdr* alleles. It is likely that the current distribution of the *kdr* alleles reflects distinct *Ae. aegypti* populations that colonized the continent. Population genetics analysis of neutral loci will help us to unravel the evolutionary routes of these resistance genes.

## Conclusions

In conclusion, pyrethroids are the most employed insecticides worldwide and the only chemical class presently allowed in long lasting treated materials, such as nets and curtains [40]. Although novel control strategies are being tested in the field, such as those based on transgenic and on *Wolbachia*-infected mosquitoes [2,41,42], insecticides will certainly play an important role for yet a long time. Knowledge of the sodium channel diversity in natural populations together with the role of each allele regarding pyrethroid resistance as well as their fitness effects are crucial for preserving the effectiveness of this class of compounds as a viable tool against *Ae. aegypti*.

## Competing interests

The authors declare that they have no competing interests.

## Authors’ contributions

Conceived and designed the experiments: JGBL, LPB, AJM. Performed the experiments: JGBL, LPB, GAG. Analyzed the data: JGBL, LPB, ASA, RVB, AJM. Contributed reagents/materials/analysis tools: RVB, JBPL, DV. Wrote the paper: AJM, DV. All authors read and approved the final version of the manuscript.

## Supplementary Material

Additional file 1: Table S1 *Kdr* allele frequencies of *Aedes aegypti* natural populations from Brazil. The CI95%* is under parentheses. Click here for file
