# Supplementary material for: Distribution and dissemination of the Val1016Ile and Phe1534Cys Kdr mutations in Aedes aegypti Brazilian natural populations
Source: Parasit Vectors. 2014 Jan 15;7:25. doi: 10.1186/1756-3305-7-25 (PMC3912884; doi:10.1186/1756-3305-7-25)
Supplement: Additional file 1: Table S1 — Kdr allele frequencies of Aedes aegypti natural populations from Brazil. The CI95%* is under parentheses. [file 1756-3305-7-25-S1.docx]

**Suplementary Table – S1**

*Kdr* allele frequencies of *Aedes aegypti* natural populations from Brazil. The CI95%* is under parentheses.

* IC95% interval is equivalent to two standard deviations, obtained by the allele frequency variance formula *p(1-p)/2n*, where *p* is the allele frequency and *n* is the population sample size.
